# An integrated geospatial modelling framework of hybrid microgrid sizing for rural electrification planning

**DOI:** 10.1016/j.mex.2025.103153

**Published:** 2025-01-07

**Authors:** Berino Francisco Silinto, Darlain Edeme, Silvia Corigliano, Aleksandar Dimovski, Marco Merlo, Christian Zuidema, André Faaij

**Affiliations:** a Department of Planning, Faculty of Spatial Sciences, University of Groningen, Landleven 1.9747 AD, Groningen, Netherlands; b Department of Mechanical Engineering, Faculty of Engineering, University Eduardo Mondlane, Av. de Moçambique km 1.5, Maputo, Mozambique; c Polytechnic University of Milan, Piazza Leonardo da Vinci 32, Milan, Italy; d Integrated Research on Energy, Environment, and Society, University of Groningen, Nijenborgh 6, 9700 AE, Groningen, Netherlands; e TNO Energy & Material Transition, Utrecht, Netherlands

**Keywords:** Optimization, Least-cost electrification, Grid routing, Hybrid renewable energy, GISEle, Geospatial planning, GISEle_V01+ (GIS for rural electrification)

## Abstract

Pursuing rural electrification in developing countries through hybrid generation systems is constrained by a lack of suitable energy modelling tools. Few tools include geographical parameters relevant to capturing specific spatial and socio-economic circumstances. Even less are openly available and find applications for rural areas of developing countries. This work presents an integrated geospatial energy modelling framework based on an extended tool, the GISEle (GIS for rural electrification) model, which aims for a least-cost energy solution. GISEle is an open-source tool supporting rural electrification planning strategies and challenges through optimal hybrid microgrid integration. The developed framework is universally applicable and explains how the extended GISEle tool can be used to become suitable for analysing decentralised hybrid generation systems within the context of rural areas of developing countries. This presented framework includes:

• Advancing the approach to proper data collection to better capture local specificities and (future) demand and reporting results in rural areas of developing countries;
• Adding the Remote-Areas Multi-energy systems load Profiles (RAMP) to improve load demand assessments, while considering the impact of electrification on growing demand scenarios;
• Linking the Soil and Water Assessment Tool (SWAT) model to allow for hydropower sizing in GISEle.

## Graphical abstract

**Figure:**
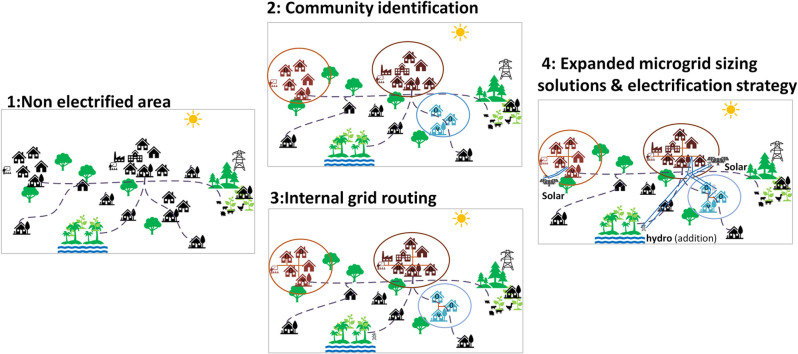

Specifications table

**Table:** 

Subject area:	Energy
More specific subject area:	Integrated geospatial energy system modelling -Hybrid microgrids
Name of your method:	GISEle_V01+ (GIS for rural electrification)
Name and reference of original method:	GISEle-GIS for rural electrification: Corigliano, S., Carnovali, T., Edeme, D., & Merlo, M. (2020). Holistic geospatial data-based procedure for electric network design and least-cost energy strategy. *Energy for Sustainable Development, 58*, 1–15. https://doi.org/10.1016/j.esd.2020.06.008
Resource availability:	• GISEle_V01 (Expanded version): https://github.com/Energy4Growing/gisele_v01. For additional data, please refer to Table 1, Table A1 (Appendix A). • Anaconda environment: available at https://repo.anaconda.com/archive/Anaconda3-2023.07-2-Windows-x86_64.exe - GISEle software installed within the anaconda environment including basic libraries; • Gurobi Optimizer version 9.5.2 build v9.5.2rc0 (win64): https://www.gurobi.com/academia/academic-program-and-licenses/ - GISEle works with Gurobi, a MILP solver optimizer. A free academic license is available upon registration with academic details/email. However, other “pyomo” compatible solvers such as CPLEX, GLPK, etc. also work; • Access and registration to the Earth Engine website: https://earthengine.google.com/ is also required if the user enables the internal capabilities of GISEle to download GIS datasets automatically; • QGIS-Quantum GIS: https://www.qgis.org/en/site/. Data processing and visualisation; • RAMP model: https://github.com/SESAM-Polimi/RAMP_multiyear. Accessed through Spyder within Anaconda or another Python environment; • SWAT tool: https://swatplus.gitbook.io/docs/installation

## Background

Rural electrification in developing countries through grid extensions risks being economically unviable [1]. An alternative is to rely on decentralised hybrid renewable energy systems (HRES), which due to improvements in renewables have become more economically attractive and viable [2]. Designing and scaling decentralised HRES options requires the use of suitable energy modelling tools. The selected tools are key for analysing potential impacts resulting from decisions taken on possible future energy system developments while considering various assumptions, scenarios and data inputs [3]. Analysing HRES as a strategy for rural electrification requires a detailed understanding of uncertainties in local energy demand and resource patterns. For such purposes, modelling tools should first consider the targeted area's characteristics related to terrain, population density and electric demands, existing energy resource potentials, and infrastructures. Secondly, they should be able to analyse the complexity of network and hybrid microgrid configuration designs including the dynamics of the integrated renewable energy sources to balance supply and demand [4]. Finally, as electrification strongly influences local socioeconomic development, proper scaling considering the possible impacts of electrification on future energy demands is necessary. Consequently, there is a need for integrated modelling approaches that allow for geospatial data analysis and optimisation of hybrid microgrids in the face of growth [5].

Few models include geospatial parameters, with even less finding application in the context of rural areas of developing countries [5,6]. Among these, is GISEle (GIS for Electrification), an open-source Python and GIS-based tool, developed for improving rural electrification in developing countries. The tool integrates state-of-the-art spatially explicit algorithms and modelling approaches, including Density-Based Spatial Clustering of Application with Noise (DBSCAN), graph theories-based algorithms, minimum spanning tree (MST), Dijkstra and Mixed Integer Linear Programming (MILP) optimisation model [7]. Combined with the mentioned approaches, GISEle, described as GISEle_V01, relies on locally tailored and openly available geospatial data to analyse population settlements and assess exploitable energy resource potentials to optimise least-costly electric grids and decentralised HRES generation and potentially on-grid connection. Previous contributions [7,8], have validated the potential of its use in providing analytical support for rural electrification planning challenges. The early version of GISEle relies only on wind and solar generating technologies and backup diesel generators and energy storage systems. Moreover, like any other modelling tool, GISEle relies on reliable local data, which may not be easily accessible in rural developing contexts [9]. Nevertheless, it lacks capabilities in sizing other promising technologies such as hydropower or biomass including no proper procedure for developing realistic demand profiles that can anticipate growth paths after electrification.

This study discusses how GISEle_V01 can be expanded to become suitable for analysing HRES integration within the diverse context of rural developing countries. In doing so, the study proposes a distinct framework regarding data collection and use of GISEle, while discussing and explaining the following methodological improvements made This study firstly explains the capacity expansion of GISEle_V01 by adding hydropower sizing capabilities to its set of wind and solar sizing technologies and linking a module for analysis of possible changing demands. For creating more representative daily load demands, this study linked the Remote-Areas Multi-energy systems load Profiles (RAMP) to GISEle. RAMP is, a bottom-up open-source python-based stochastic load demand generator [10,11]. By considering differences between user classes, the number of user classes and the use of various assumed appliances per user, RAMP can produce detailed load profiles useful for GISELe. Hydropower sizing benefit from linking GISEle to the Soil and Water Assessment Tool (SWAT) [12]. SWAT is widely used to estimate river flow rates in complex and limited data availability watersheds. While RAMP did not require explicit changes to GISEle itself, using SWAT in this study did require some customisation to be made within GISEle microgrid sizing procedure structure. These meant to enabling GISEle with capabilities to import river flow discharge estimates from SWAT and further assess the hydro resource potentials and sizing the hydropower capacities in the targeted area. Both RAMP and SWAT require tailored and publicly available geospatial data, linked to the second main contribution of this study. That is, this study embeds GISEle and its extensions in a framework consisting of five methodological steps that make it suitable for analysing the integration of HRES in any rural setting. These steps explicate how various datasets can be accessed and fed into GISEle and how its considered features logically link. In doing so, these steps also identify opportunities for field surveys to enrich datasets and help identify impacts of electrification on growing demand scenarios. Finally, the framework provides a ready-made step plan to support rural electrification planning challenges.

## Method details

### Conceptual modelling framework and procedures

**Fig. 1:**
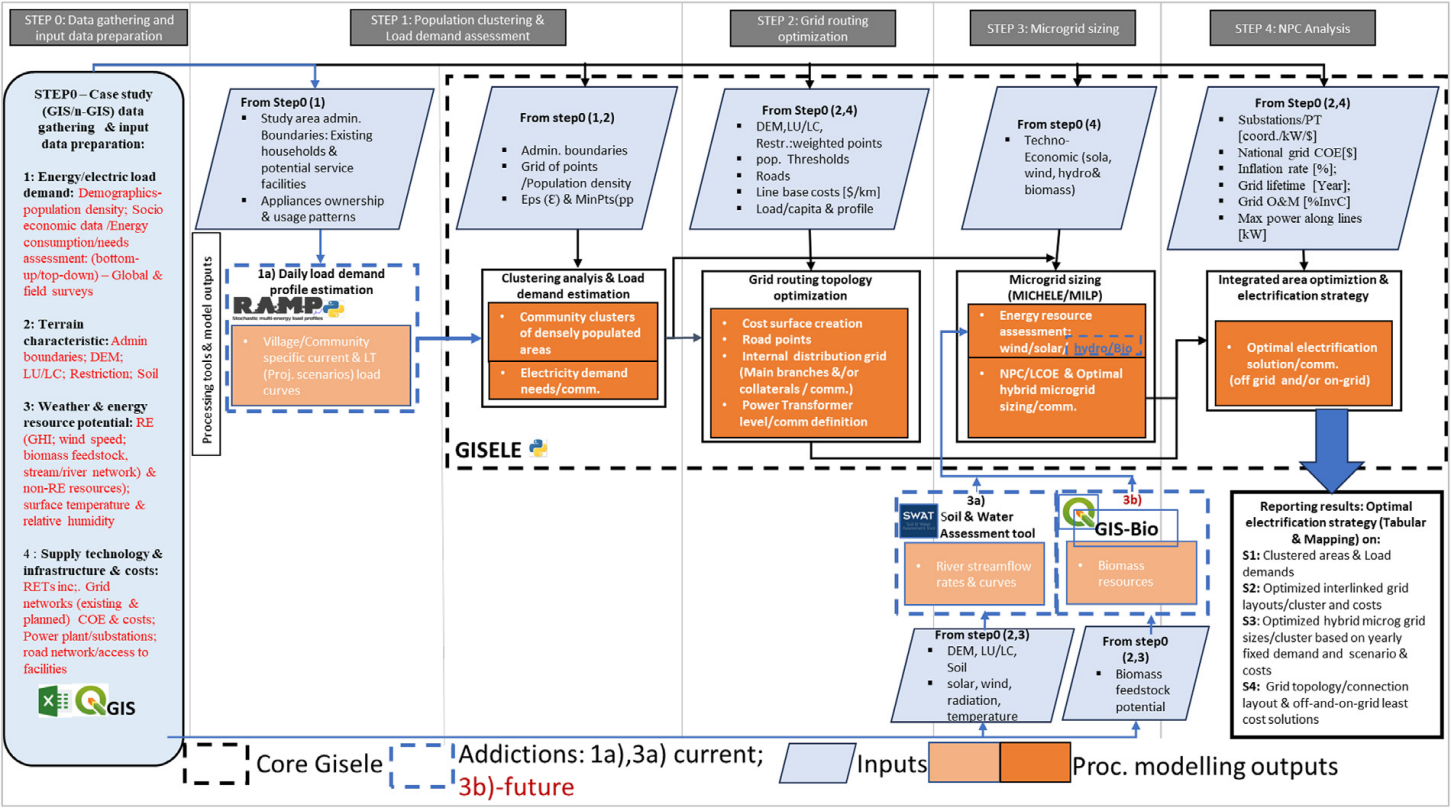
Schematic flow chart of the integrated modelling framework rooted within the GISELE_V01 interface.

The proposed modelling framework consists of five sequential steps implemented within the GISEle environment as shown in Fig. 1. The first step involves input & geospatial data processing (step 0), which takes place before using GISEle. Steps 1–4 all rely on the use of GISEle, starting with clustering and demand assessment (step 1), grid routing (step 2), microgrid sizing (step 3) and NPC analyses (step 4). This section discusses all steps in more detail while highlighting both the consideration of data relevant for rural areas of developing countries (in step 0), and the capacity expansion of GISEle executed in this study with the inclusion of the RAMP and SWAT modules. Each integrated modelling procedure has specific sub-steps and modelling formulations/algorithms for specific rural electrification problem-solving.

**Fig. 2:**
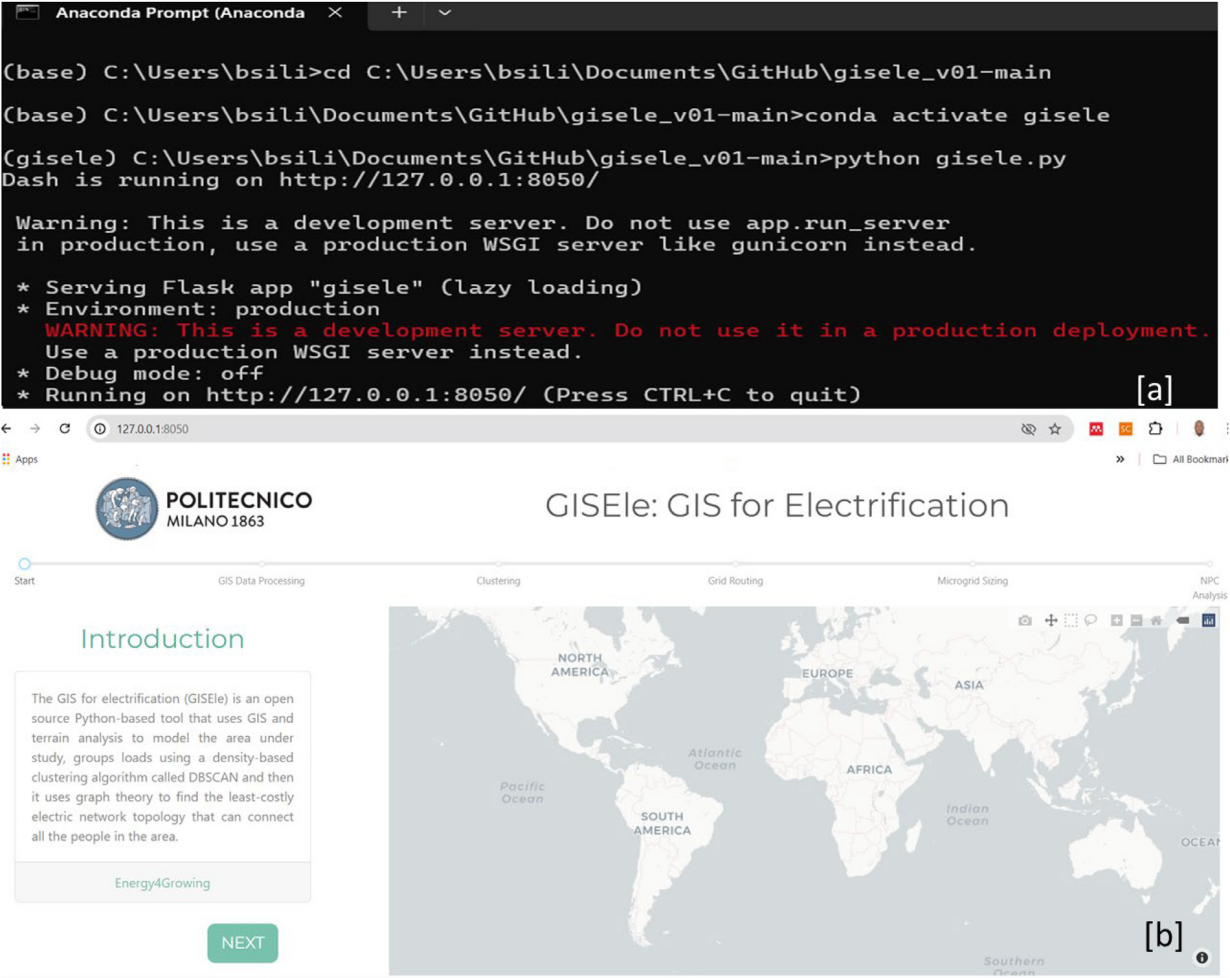
GISEle graphical user interface showing the integrated modelling procedures tabs (b) which can be visualised over a local internet web browser after activation and successfully established a virtual connection server within the anaconda (a) or another Python environment.

The core mentioned steps from 1 to 4 can be visualised within the GISEle graphical user interface (GUI), represented by their respective tabs as shown in Fig. 2. In the next paragraphs each procedure is stepwise explained:

### Data gathering and preparation: geospatial data processing (step 0)

Reliable information and data of the intended area to be electrified is required. In this framework, key datasets include at least the administrative boundaries, socio-economic and demographical data including population density, energy resources potentials and related technologies, terrain characteristics, and existing and planned road and electric grid infrastructure of the targeted study area. Some of these datasets can be extracted from national or international database repositories, but acknowledge conducting field studies is useful to assess actual circumstances (the ground truth). Fieldwork is an important task for data validation and gaining a more detailed understanding of the demographics, socio-economic conditions, and specific user needs. Notably, this allows for improved estimates of load demands and their respective profiles, while accounting for the uncertainties of possible evolving demand over time. This approach was applied in the extended GISEle tool described in this study, for real rural study cases in Mozambique, which also produced the key inputs used and further explained the extensions made for its validation. This approach helps to get insights into the process for developing load demand-based scenarios about future energy prospects. Specifically, the approach relied on interview-based field data collection using questionnaires both locally and through online data survey platforms (Kobo toolbox^1^) where the collected data was readily and digitally made available.

**Fig. 3:**
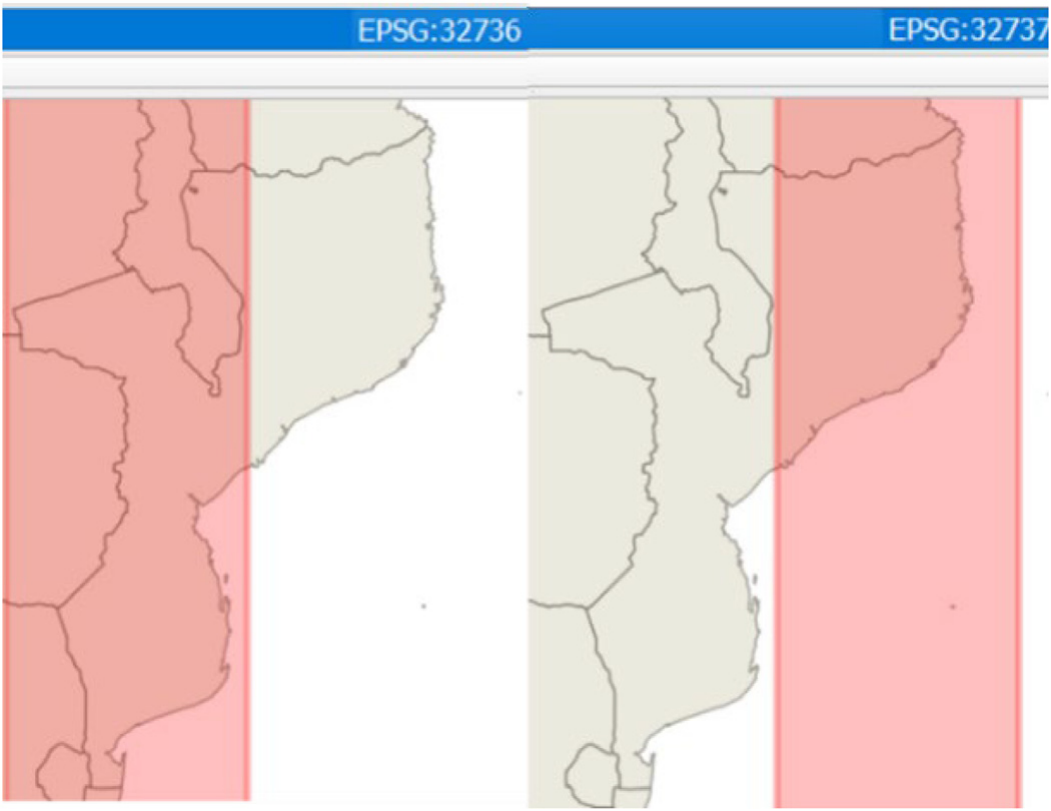
Representation of predefined UTM zones and CRS codes for Mozambique (coloured area in red).

The Microsoft (MS)-Excel and QGIS^2^ environments are the main pre-processing tools for preparing and unifying datasets into workable data formats (.csv or .shp GIS raster or vector layers^3^) as required to be imported and processed within GISEle procedures. The used geospatial data files need to be georeferenced and reprojected into a single Universal Transverse Mercator (UTM) zone and coordinate reference system (CRS) which geographically best fits the targeted area [13]. Using projected files with different UTM and CRS codes will not converge, and turn the procedures into errors. For instance, Mozambique is covered by two UTM Zones i.e., WGS 84: UTM Zone 36S (EPSG 32,736) and 37S (EPSG 32,736) respectively as shown in Fig. 3. The user should choose which type to work with based on its preferences and considering the minimisation of distortion effects as explained in [13].

Within GISEle, each step relies on its model runs that are sequentially independent from the runs that are part of the following step. Each step thus generates an output file that is required in the subsequent step to run; i.e., step 2 will run after step 1 successfully generates its outputs. The key input datasets related to all independent steps of the framework are presented in Appendix Table A, including possible data-gathering sources, mostly for the case of Mozambique. This subsection continues with describing the different modelling procedures and the corresponding input/output data.

a) Input data preparation

Before starting the GIS data analysis step, the grid of points.csv input parameter that initialises the GISEle model runs is previously created using a specific input preparation Python script/algorithm [14] and the above-mentioned processed datasets. The grid of points spatially aggregates all the attributes that characterise and are used to model the case study area to be electrified. These attributes in GISEle_V01 include population density, elevation, slope, land cover, protected area, available road distances, and the river stream flow. Table 1 lists different data for creating the file grid of points and their respective global data sources. However, better and updated datasets may be available in the study area, for example, due to relevant country databases.

**Table 1:** Data inputs for the creation of grid points and cost surface files.

Input parameter	Type	Resolution (m)	Sources
Administrative boundaries	Vector layer (polygon)		[15,16]
Population and density distribution	Raster layer [people/m^2^]	100	High-resolution settlement layer database [17,18]
Land use/cover/ GLC 2000	Raster layer 22 land cover types	500	[19]
Digital Elevation Model (DEM-SRTM) (slope/elevation)	Raster layer [m]	30	[20]
Global River Network (HydroSHEDS)	Vector layer (Polyline)		[21,22]. https://www.hydrosheds.org
Road network	Vector layer	polygon	https://www.openstreetmap.org/
Protected/Restriction Zones	Raster-Vector	Polygon	[23,24]

**Fig. 4:**
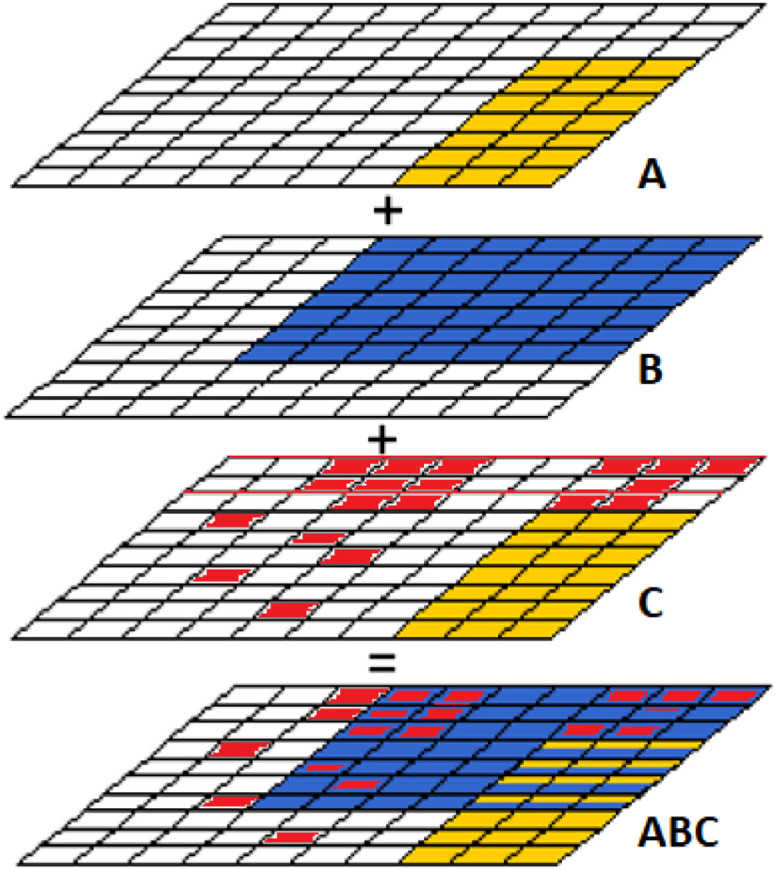
Example of an overlayed study area (ABC) vector/raster layers with the same cell size resolution and CRS.

The population density and distribution are the most important input information for performing the cluster analysis in Step 1. However, since each raw dataset has its specific format type size and resolution (Table A.1 - Appendix A), the desired working resolution must be defined (eg. 100 × 100 m), acknowledging that a higher resolution entails higher computational efforts required. Fig. 4, illustrates an example of a layer aggregation (ABC) after resampling and overlaying different layers. In this process, the assessed territory is subdivided into a regular grid of pixels, and the centroid of each X, Y^4^ square pixel size is spatially assigned to the different characteristics of its surrounding grid cells.

**Fig. 5:**
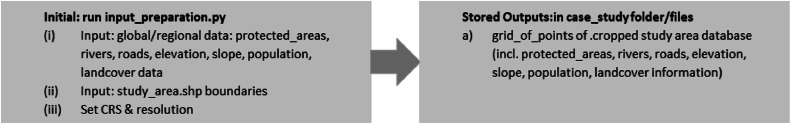
Summary of Python procedure to create “grid of points files of the study area.

The script/algorithm for developing the grid of points has been updated (fixed issues related to library updates) in this study; the process run is summarised in Fig. 5.

b) Geospatial data processing

The GIS data analysis process starts after creating and loading the grid of points, setting the land cover, the working CRS, and the resolution. The procedure loads these inputs to generate and store reprojected datasets (in .csv or .shp file formats) on roads (edge route and measured distance node layouts) including population density, elevation, and weighted points. The latter represents a weighted^5^ (W) raster layer computed in a weighting modelling strategy [25,26] so that the specificities in each pixel or point of the terrain are spatially expressed as a unitary (n) penalty factor ($Pf_{i}$), that in terms of base costs (cost surface), is cumulatively summed up (from 0→n). This factor represents the degree of difficulty in deploying an electricity line imposed by the specific topology of the terrain (such as distance to roads, slope, forest, etc.) over the deployment area [7]. Mathematically W is expressed by Eq. (1).

(1) $$W=\;\sum_{i=0}^{n}Pf_{i}\;or\;Pf=1+\;\sum_{i=0}^{categories}Penalty_{i}$$

Table 2 reports the criteria and considered coefficients in this procedure to calculate the penalty factors (Pf). Moreover, the Pf also expresses the level of accessibility of the terrain (geographical data point) for building electricity lines [25].

**Table 2:** Assigned penalty factor values according to the different land use/cover types that are further used for determining the cost surface indexes [7].

Category (Constraint factor)	Road distance (m)			Land cover			Fault/slope	River		Water bodies& Lakes		Protected areas (Cultural heritage sites; Vegetation coverage (natural parks, meadows and trees)	
Type	<100	>100<1000	>1000	Grass-Open forest	Tree cover shrubs	Closed forest	–	Yes	No	Yes	No	Yes	No
Penalty [7]	1	Linear	6	1	2–4	5–8	Exponential 1	9	0	10	0	99.999	0

**Fig. 6:**
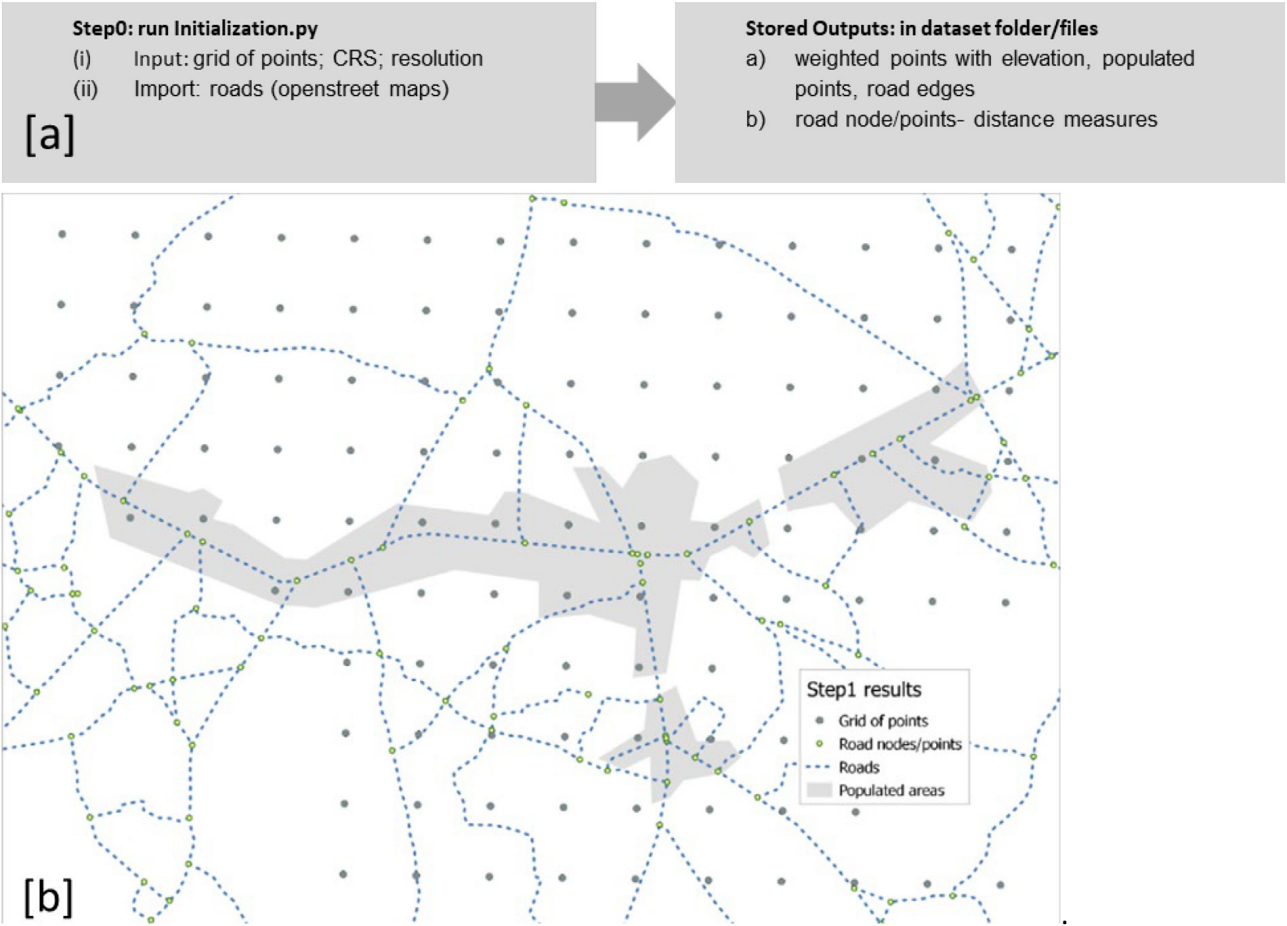
Example of a geospatial terrain data analysis and weighted strategy (a) step 0 model procedure run input/output and (b) grid of points (including the weighted graph) and road and measured road nodes (distances) also capturing high populated settlements.

Further, this factor is applied in the grid routing procedure (Step 2), which aims to optimally design the grid routing and estimate the line length and costs (cost per kilometre multiplied by the Pf) to deploy grid lines across each pixel covering the terrain. For instance, the weighted costs increase with a higher distance from the road, a higher slope and crossing extreme environments such as rivers, dense forests, etc. In this study, the type of terrain is defined based on the GLC2000^6^ project [19], and a maximum penalty factor of 10 is assigned for water bodies (Fig. 6).

### Population clustering and load demand assessment (step 1)

**Fig. 7:**
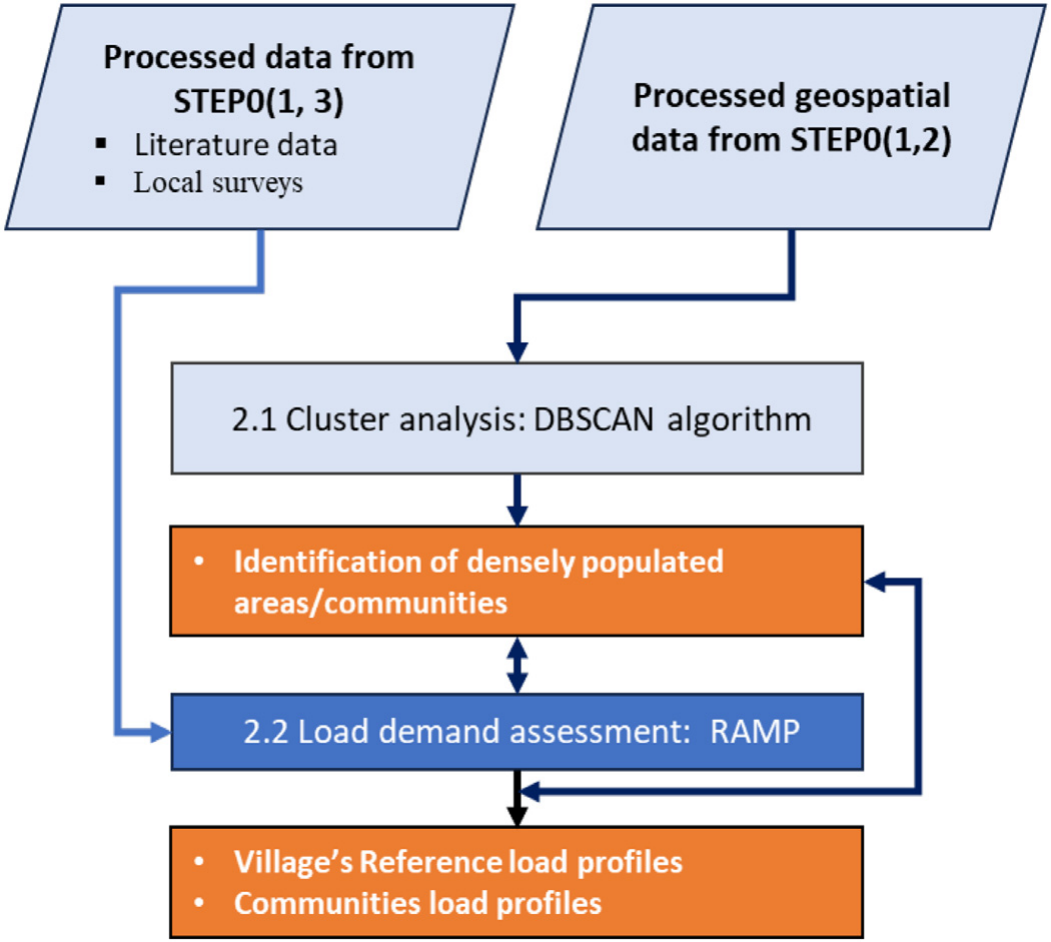
Integrated flow chart of cluster analysis and demand assessment procedures.

An effective rural electrification strategy requires a detailed assessment of the characteristics of population and their energy needs. To do so, the starting point is the identification of existing rural population settlements and further grouping them into clusters of communities to be electrified with the same electrification strategy. Subsequently, load profiles are estimated for each of the identified cluster communities. GISEle_V01, however, is less sophisticated when estimating the load profiles to be allocated to these clusters. In response, GISEle_V01 is extended by linking it with an external tool, the RAMP model. RAMP allows for estimating stochastic load demands and related profiles. The generated load profile serves as a study reference and is loaded into GISEle where internally the procedure estimates the energy needs and power pick values for each cluster community. Extracted from the main framework of Fig. 1, the flowchart in Fig. 7 indicates the position of RAMP in the extended GISEle framework.

a) Population clustering

Step 1 starts with population clustering where population settlements are spatially identified using clustering analysis^7^ techniques. For clustering, GISEle relies on the DBSCAN algorithm [27], which identifies and groups densely populated points into communities to be electrified. The DBSCAN algorithm is most suitable for complex geospatial applications. DBSCAN requires low computational efforts compared to common clustering algorithms. Some examples are, the Euclidean distance K-means or K-methods that employ hierarchical methods and that require the final number of clusters and data similarities as inputs [27]. Those do not apply to complex rural developing regions' contexts [8]. The DBSCAN can automatically detect and create/agglomerate arbitrary non-convex shaped clusters, and identify the exact extension of the high population density area including the outliers or points out of any cluster (the “Noise” element) [8], that represent sparsely located households, a real characteristic of rural communities.

**Fig. 8:**
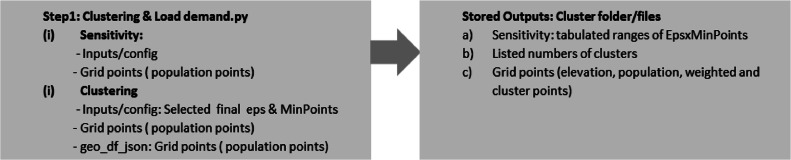
Summary of clustering analysis procedures.

In DBSCAN, clusters are built on two key input parameters: (i) MinPts - minimum number of points/people, representing a threshold set to be discoverable to form a cluster within a given radius distance^8^ (ii) Eps (ε) epsilon. This procedure uses as input the populated points (n observations associating population points to be clustered) within the previously generated and imported weighted file output file (step 1, stored output file, Fig. 8) according to the associated number of population and MinPts indicate the minimum size of a community to be considered for electrification. Both MinPts and Eps parameters can be determined by Eq. (2), where the cluster density is approximately equal to the average population density.

The detailed DBSCAN algorithm and flowchart of the implemented procedure are discussed in [7]. The DBSCAN pseudocode is shown in Algorithm 1 including a summary illustration of the clustering procedure in Fig. 8.

(2) $$\varepsilon =\sqrt{\frac{MinPts}{\rho *\pi }}$$

**Table:** Algorithm 1. DBSCAN.

Choosing the best combination among MinPts and ε clustering parameters can also be informed by performing a sensitivity analysis (an enabled feature in the GUI interface). The sensitivity analysis involves several DBSCAN runs, performed under four evaluation/decision indicators: (a) number of resulting clusters, (b) percentage of clustered people in the area, (c) percentage of clustered area, and (d) ratio between number of people and total clustered area, which helps relate with a defined electrification project's goal. For instance, in economic terms: a decision may be to reduce the size of clusters or the high ratio of people/area, thus implying a reduction of cable lengths and costs; in technology terms: to cover the entire study area, etc.) and therefore resulting in one or several clusters distributed in the study area [8,28].

b) Load estimation

**Fig. 9:**
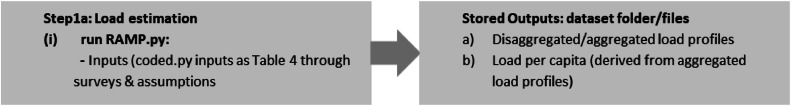
Summary of RAMP procedure to estimate load demand.

Having identified and clustered the highly densely populated areas, each cluster's populated points are also used to estimate the energy load demands. Within GISEle_V01, the load demand profile is not computed. In response, GISEle_V01 was interlinked with the RAMP model, where energy needs and its related pick power values, are modelled using reference load profiles externally generated through it (Step 1a, Fig. 9). The RAMP model is an open-source python-based (non-GIS-based) model developed at Polytechnic University of Milano [10,11]. RAMP models stochastic load profiles based on three input parameters: i.e.; (i) user class type/name, (ii) number of users/class and (iii) owned appliances per user in each user class, including their use cycle/time and functionality dimensions. This information can be derived from field surveys, literature and expert assumptions. Table 3 describes each of the input parameters required in the RAMP tool. These parameters are coded in Python as exemplified in Fig. B1 (Appendix B). The input reference loads are further associated to a proxy number of existing households of each identified community cluster and then scaled up to the studied area.

**Table 3:** Main input parameters considered in RAMP model.

Parameters/ Dimensions	Description	Unit (range) measure
Userj	Category name of each User class	User Type
Nj	Number of users within a specific Userj that owns specific appliance(s)	0-n
Appliance	Name/type of appliance owned by each user in a class j	Appliance type
n_ij_	Number of appliance types i within class j	0-n
P_ij_	Nominal power absorbed by specific appliance (s) ij	0–20k [W]
fw_ij_	Number of functioning window times: periods during the day each appliance can be switched on	1–3
W_f,n_	Start and end times of appliance's use	0:00–23:59
Rfc_ij_	% of random variability of daily functioning time allowed in a defined functioning cycle (mainly for thermal appliances)	0–100 [%]
ft_ij_	Daily functioning time: daily total time the appliance is used (kept switched on)	0–1440 min
fc_ij_	functioning cycle: minimum time appliance ij is kept on after switch-on	0–1440 min
Rfwij	Percentage/probability that the appliance is occasionally used in a single day	0–100 [%]
	Constraint factor for the appliance usage specifically on weekday or weekend periods	We/wd/none

For making use of the RAMP model in the context of GISEle, the specific user(s) of class type (User_j_) can be a group of households (e.g. discretised by building types/income/owned appliances, etc.), public (offices, school, hospital, etc.) or productive facility (shops, processing industries, etc.). Then the number of users (N_ij-jn_) of each type within (User_j_) are identified followed by their owned type (App_jik-jik-jim_) and their associated number(n_ij_) of electrical appliances including their rated power (P_ij_), frequency/functioning time/hours/day (ft_ij_/h_funct_, cycle (fc_ij_=min on) and possible functioning windows (W_f,n_)/periods within (ft_ij_/h_funct_) as expressed by Eq. (3). The detailed modelling procedures are referenced in [10] and accessible on the GitHub platform (https://github.com/SESAM-Polimi/RAMP/tree/MultiYear_Load, n.d.).

(3) $$Tot_{w}=\sum_{n=1}^{Num\;Win}W_{f,n}\;\left[h\right];\;h_{funct}\leq Tot_{w}$$

Therefore, the resulting estimated load curves are combinations of each appliance's usage patterns (equivalent power (Peq.App) when switched on/off during the day along the functioning $h_{funct}$periods as given by Eq. (4).

(4) $$P_{eq,App}*Tot_{w}=P_{App}*h_{funct}*N_{App}$$

In each model run and user class, RAMP creates 365 daily load curves/year through a random variability of simulations. Computational burdens to run the model may be an issue, depending on the input coding details, such as the size of the surveyed service uses versus the number of appliances in a single user class type and/or integrating multiple user service details. This may lead the processing time to take hours or even several days to generate its final outputs. Thus, for using the RAMP with high amounts of data it is advisable to run in high-performance computers.

The level of detail RAMP can process and model implies that proper load profile estimates also require a strong underlying dataset. Limited data availability and the reality that many households in developing countries are still to be electrified are both arguments for utilising on-site data collection methods such as surveys or inquiries among the population of the communities. Literature information and national population census may well prove useful, but a keen understanding of the local realities and expectations on what households, businesses and other organisations may use typically requires an understanding of the local circumstances. The data gathered from surveys to actual users include questionnaires collecting information on energy access to services and consumption types, satisfaction and affordability levels as well as number and type and related power ratings of owned electric appliances, usage frequency and windows, etc. This information can be provided by experienced villagers, key government officers and community leaders including some households. Similarly, they may help to translate needs and behaviour of existing users into what newly electrified users may need. Surveys and interviews are further important sources to assess the village's socio-economic situation and future development plans. Combined with other literature [10,29] such as the World Bank Multi-Tier Framework-MTF [30] on the most currently used electric appliances and services, these surveys and interviews thus help make more realistic demand estimations.

The estimated electrical load profiles and their associated Load per capita (LpC)value are the main input parameters imported in the following grid routing procedure (Step 2). It is assumed that the load profiles and the estimated power peak values can be sustained by a suitable grid solution with considerations that LpC multiplied by coincidence factors is sufficient to size the grid lines and their costs [31,32].

### Grid routing optimisation (step 2)

The grid routing procedure uses a geospatial topological approach to design the medium voltage (MV) electricity distribution grid layout interconnecting each cluster's populated points. Combined with the weighted grids-cost surface maps, the procedure provides a reliable least-cost grid solution considering a hierarchical structure comprising of main branches and collaterals^9^ [8] and the shortest path analysis including the possible location of feeders/substations (eg. pole mounted medium or low voltage power transformers) [7]. The internal grid topology connections are based on GISEle's embedded graph theory algorithm. This algorithm transforms the previously determined weighted points (Pfs) (in step1) into a cost surface factor-based weighted graph [26]: G = (V, E), with “V” being the vertexes (pixels’ centroids) while “E” is the edges of node/ load connections. To each edge connecting two vertices/points (u&v) a cost $C_{u,v}$ for deploying an electric line connecting both is assigned. It is worth noting the cost of deploying electric lines is assumed to be directly proportional to the line length by the terrain characteristics (cost surface’ weights). A detailed explanation of the algorithm and its functionalities can be referred to in [8]

**Fig. 10:**
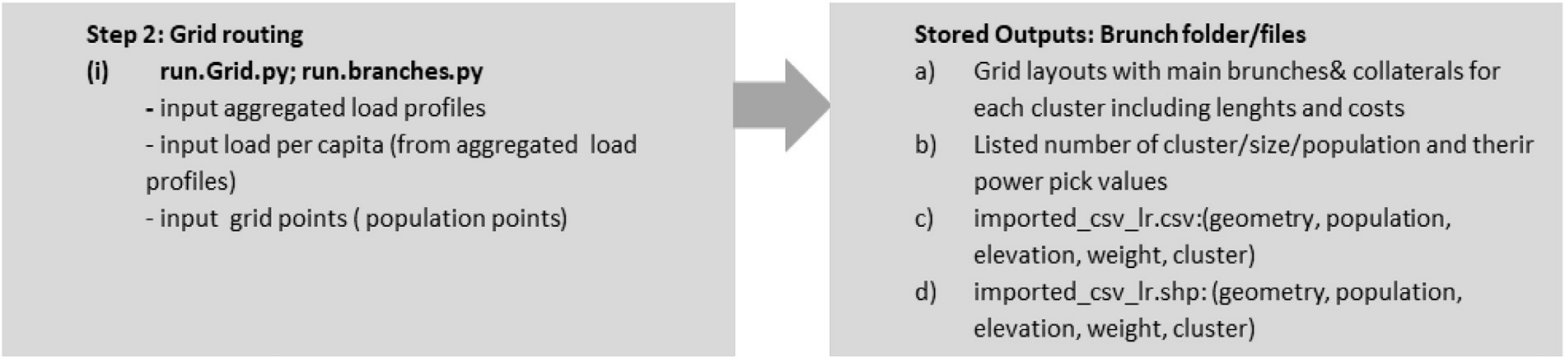
Summary of grid routing procedure model run and key input/output parameters.

**Fig. 11:**
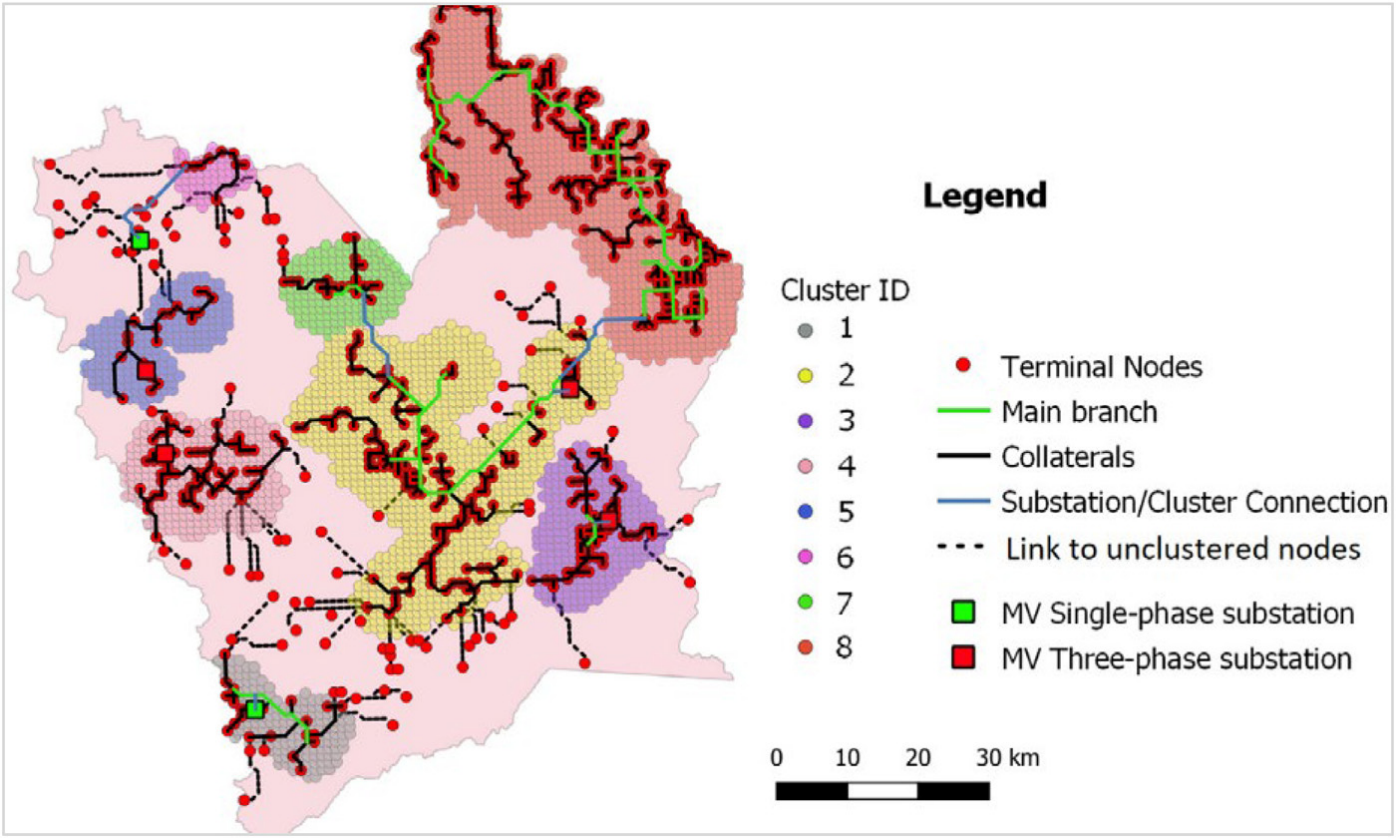
Example of implementation of main branch and collaterals approach including the options for full electrification [8].

The grid routing algorithm uses each cluster's aggregated load points to internally derive their respective power peak levels considering the pre-computed reference load profiles of per capita values [7]. By enabling or not the branches' functionality (including collaterals), it designs the optimal internal grid routing layout to electrify each cluster while determining their power peak levels. In addition to the general grid layout generated for each cluster, the user can set the procedure to include full electrification. In this way, after designing the grid layout for each cluster community, the procedure further expands the connections to populated points outside each clustered area. Finally, based on the generated grids, it is possible to define the requirements of medium or voltage grid line types for allocating their corresponding power transformers/substations. Fig. 10 illustrates the process of applying the grid routing procedure while Fig. 11 the output from the implementation of full electrification including main branch and collaterals approaches.

As the final output solution, the algorithm computes the costs sustained for the electrification process, both in terms of the power generation portfolios for off-grid systems later discussed in microgrid sizing- Step 3″ as well as in NPC analysis Step 4, for the network infrastructure required to on-grid (national grid) connections. While doing so, any available data of the electric grid at the nearest primary substations/power transformers should be loaded in, to evaluate the possible national grid connections (including between clusters) under consideration of their distance and voltage levels according to each cluster. The estimated electric needs are further used to optimally define and size the dispatching logic of potential available generating sources that fulfil such needs in step 3. The upgraded grid routing modelling structure is further explained in [7,8].

### Microgrid sizing (step 3)

**Fig. 12:**
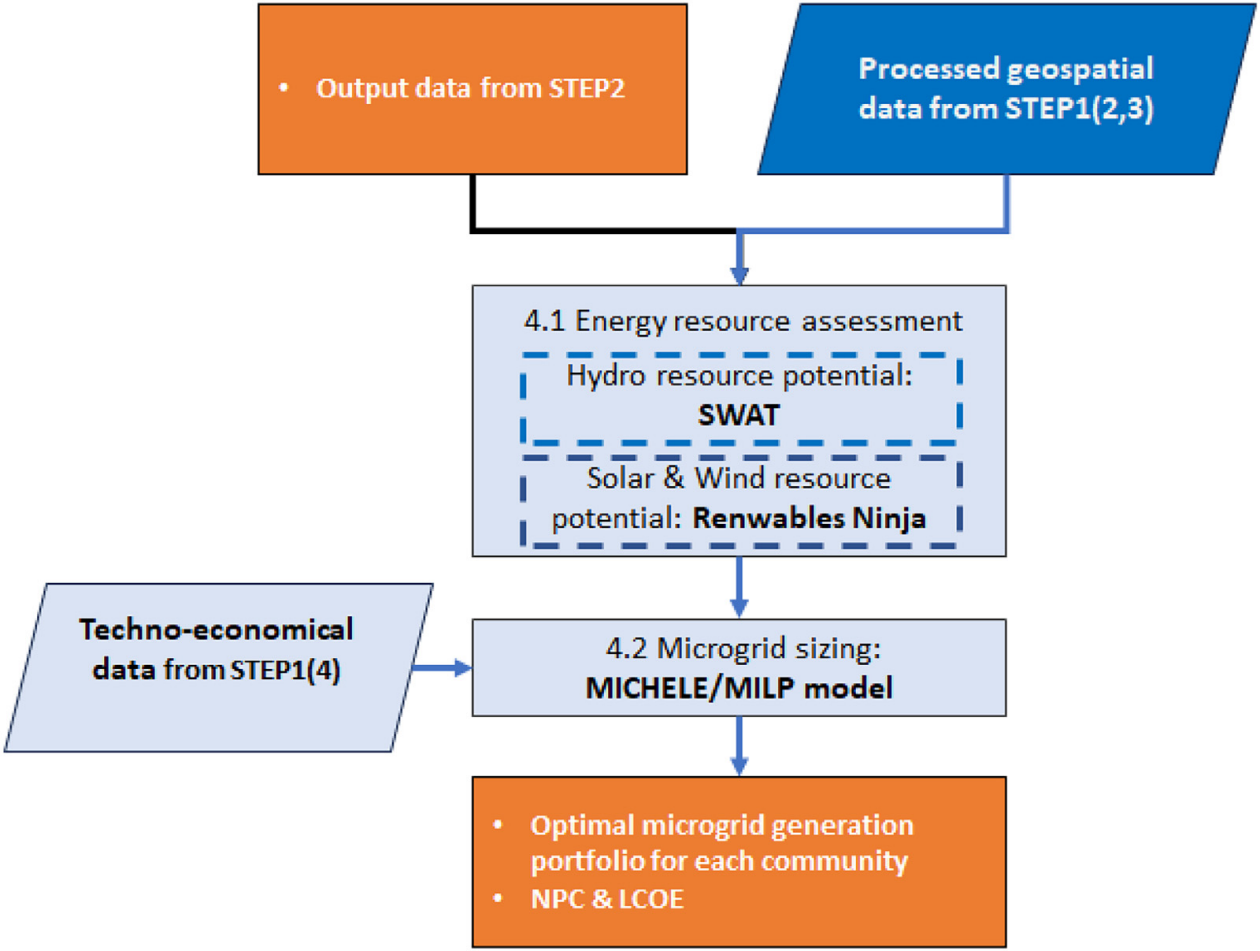
Flowchart of the microgrid sizing procedure.

The microgrid sizing procedure starts with an assessment of the renewable energy resource potential available in the study area which also includes diesel generators and storage systems (step 3a). Subsequently, it is linked to the estimated load demands in an optimisation process. The microgrid sizing seeks the optimal techno-economical hybrid microgrid configuration. In this procedure, a Mixed Integer Linear Programming (MILP) model embedded in GISEle is applied to identify and generate the optimal electrification generation RE technology portfolios which in a defined project timeframe can satisfy the previously estimated loads. Fig. 12 presents the flow chart of the microgrid sizing modelling procedure and their related sub-steps described as follows:

a) Energy resource assessment

GISEle_v01 is developed for estimating the availability of renewable energy resources within the geographical boundaries of the study area targeting wind and/or solar energy. Both availability of wind and solar energy resource assessments are internally computed considering the study area's geographical features (user-defined). Estimation is based on an application programming interface linking to an open-access database [33]. The estimation procedure automatically starts downloading hourly/unit PV power profiles (solar irradiation (GHI)) and wind resource potential time-series datasets.^10^ These estimates are derived from combined satellite data, local measurements and reanalysis techniques [34,35]. Then the computed hourly power profiles are further reshaped to consider only a few typical days of a year to ease its application within the microgrid sizing procedure [28].

The suitability of this tool for analysing and sizing HRES in rural areas of developing countries benefits from expanding the range of technologies to be included. For this study, a key expansion is the addition of a module allowing for the assessment of hydro resources and power potentials. For doing so, the SWAT+ model is added to GISEle as an additional module while algorithms within GISEle_V01 have been customised to be able to consider sizing hydropower technologies in the optimisation process. This feature is added within microgrids sizing module algorithms, specifically for interlinking the generated hydropower potential derived from river flow rate discharge outputs within the SWAT+ model as input data. The SWAT model is a physical hydrological model based on water balance principles and simplifications of the hydrogeologic cycle that combines geospatial data on elevation, land cover, soil and weather patterns to allow for a detailed description of the different processes contributing to runoff formation and river flow rates in large and complex watersheds [[36], [37], [38], [39]]. This tool has been widely applied in hydropower projects [[40], [41], [42]] and it is applied in this work. The addition of SWAT to GISEle will not only help the tool be more useful for analysing additional sets of hybrid technology configurations but is also helpful to better estimate hydro resource potentials that often hamper the development of hydropower projects in most developing countries’ watersheds [42].

**Fig. 13:**
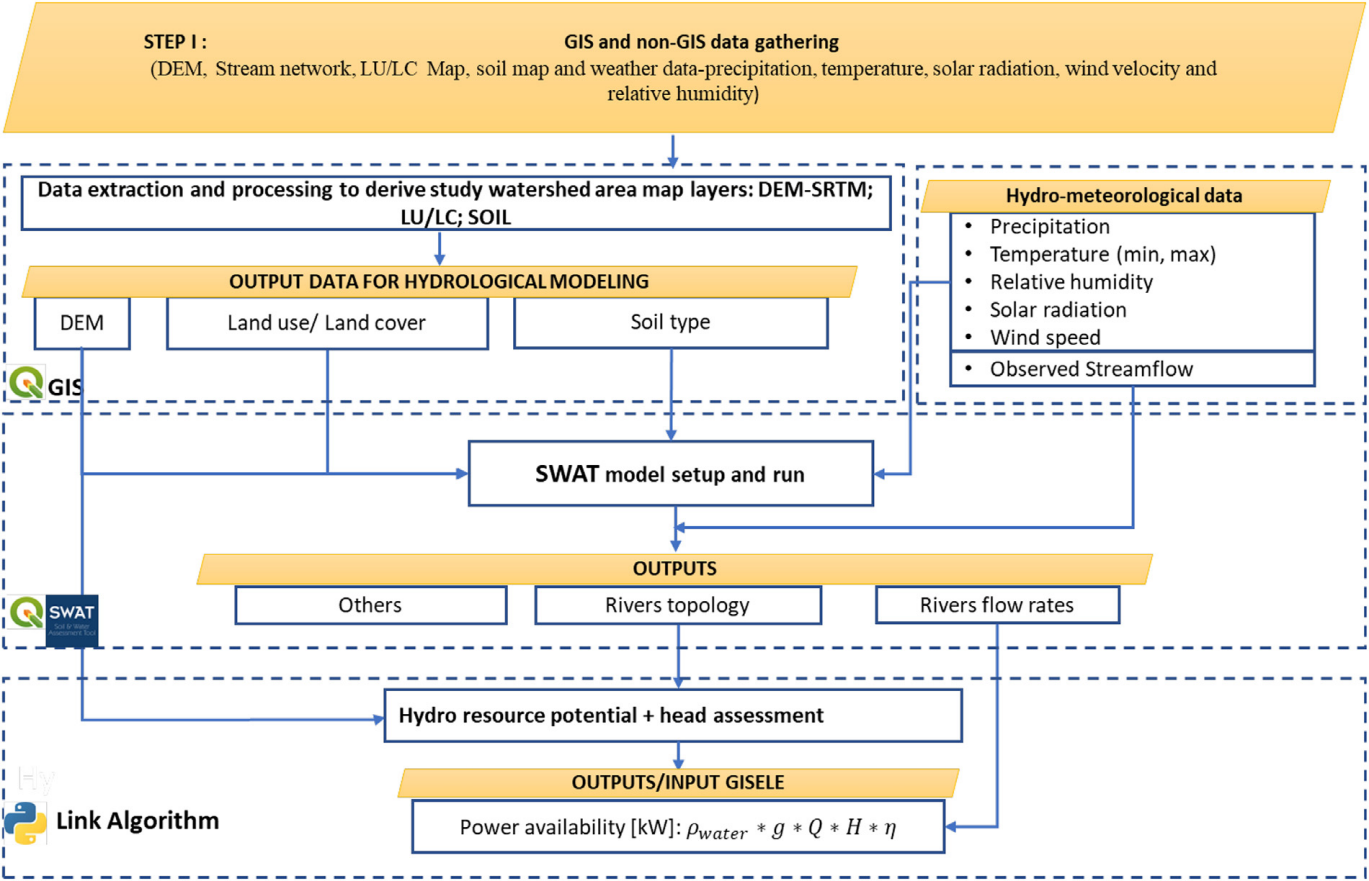
A workflow of the SWAT/hydrologic model to estimate river flow rate and the output to link with the improved GISEle_V01+ for hydropower potential assessment.

The SWAT model procedure starts by choosing the watershed area of the hydrological basin and the outlet point (connected with and related to the target study area) which includes of all water streams flowing from its river tributaries. Secondly, the watershed area is subdivided into sub-basins. For each sub-basin, the accumulated river flow rate is computed. Further, through an iterative procedure the river flows are combined with climate data to finally provide information on the hydrology patterns of the basin area including the total river flow discharges. This process is followed by a data validation process. Then the latter, depending on the desired model configurations the river flow discharges are provided at daily, monthly, and yearly averages covering the years with available climate data. The detailed activities within the various procedural steps for inputs to running the SWAT model are summarised in Fig. 13 and explained in [43,44].

The integration of SWAT within GISEle is accommodated by developing a python-based algorithm to link the river flow discharges (water runoff estimation) output information from SWAT model with the new integrated hydro procedure in GISEle. The algorithm imports river discharge data and within the watershed area combines it with the available heads^11^ along the river path to compute the hydro resource potential considering the average power availability of each closest river associated with each cluster community, set above a certain threshold. The final output is the average monthly power profile estimated using Eq. (7) associating with several hydro turbine types (chosen among a set of possible sizes with costs varying according to economies of scale). The coding details on the related customizations and updates can be found at https://github.com/Energy4Growing/gisele_v01.

(7) P(t)=Q(t)*ρ*g*H*η

where Q(t) is the average river flow rate at a monthly basis [m3/s], g is the gravitational acceleration [9.81 m/s^2^], ρ is the density of water density [1000 kg/m^3^], H is the available net head [m] and *η* is the hydropower efficiency (Nasir, 2014).

b) Microgrid sizing and analysis

**Fig. 14:**
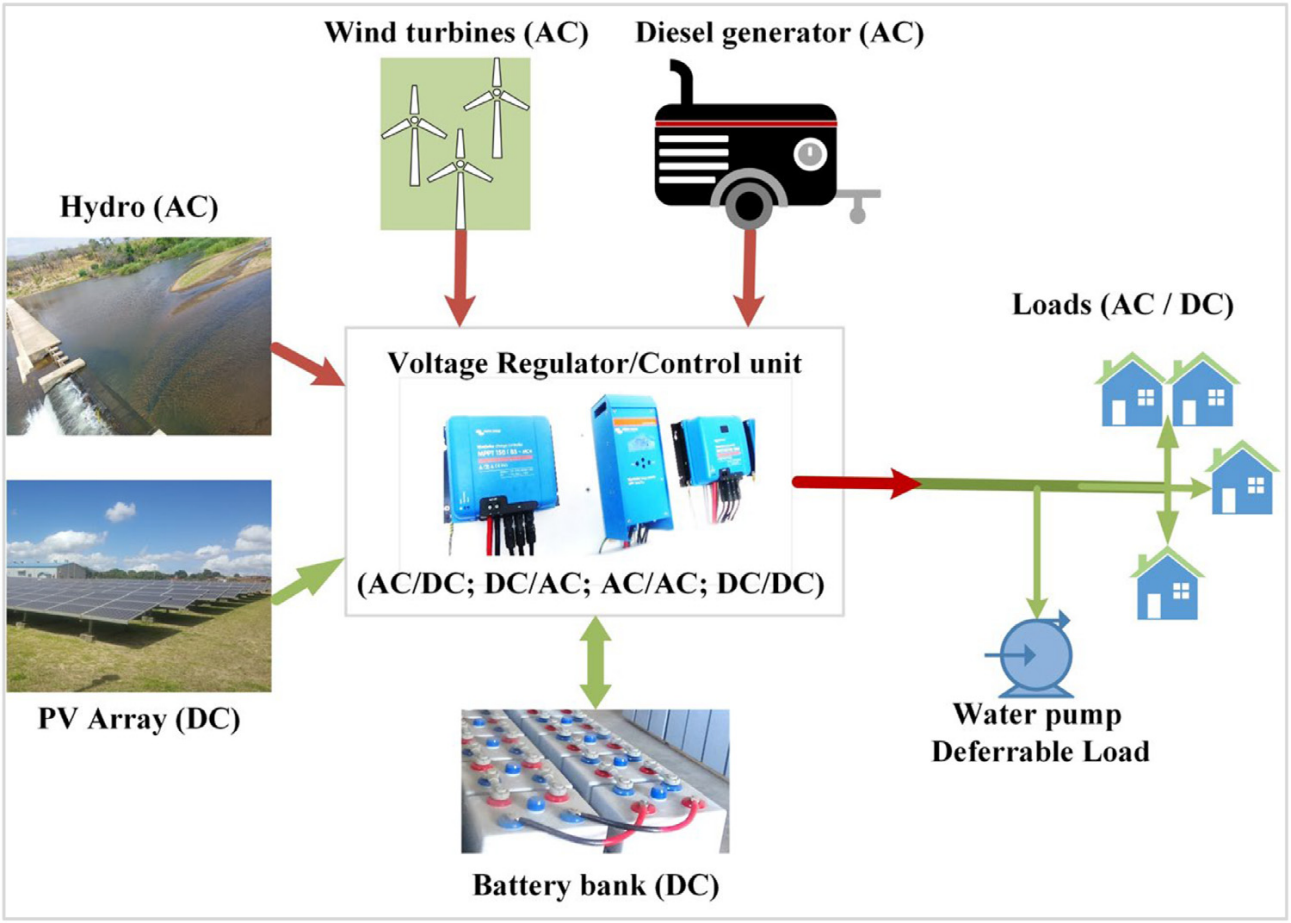
A typical schematic diagram of hybrid microgrid architecture (Source: [6]).

The microgrid sizing procedure within GISEle seeks the optimal techno-economical hybrid microgrid configuration, by combining the estimated load profile and related demand scenarios with available RE resource potentials. A typical hybrid microgrid configuration modelled in the expanded GISEle version is illustrated in Fig. 14. It includes solar photovoltaic (PV), wind turbines (wt), hydro turbines (ht), diesel generators (g), and battery energy storage systems (BESS) technologies coupled at the AC busbar to supply the required load demands. The hydro turbine is directly associated to the SWAT model's river flow outputs and its selection in the optimisation process is constrained by the availability of exploitable hydro resource potential within a maximum radius distance (set by the user) between the assessed rivers to each identified cluster community. Moreover, the costs for energy production also include the electric line length for connecting the community grids.

The model simulations are run over the project lifetime until the generation portfolios are computed. However, in the face of computational burdens, there is a possibility of selecting only a reduced number of typical days “Nd” for each year, from which the annual RES and typical daily load profiles are randomly extracted and resampled accordingly also to account for possible growth scenarios. The Net Present Cost (NPC) expressed in Eq. (8), is the objective function to be minimised. This comprises the “initial investment costs of components ($IC_{i}$), operation and maintenance ($O\&M_{i}$) costs, replacement ($RC_{i}$) costs and salvage values ($SV_{i}$). The latter represents the worth remaining in the system components at the end of the system operation period. Each of the available generating technologies is represented by sets of technologies “i”(pv, wt, ht, dg, bess) of different types, specifications, costs, and number of generators

(8) $$minNPC=IC_{i}+O\&M_{i}+RC_{i}-SV_{i}$$

Then, the optimal size of the available technologies and storage systems is selected to meet the estimated peak demands of each community cluster, through an accurate modelling structure of all components and a multi-year planning accounting for the degradation of the assets.

**Fig. 15:**
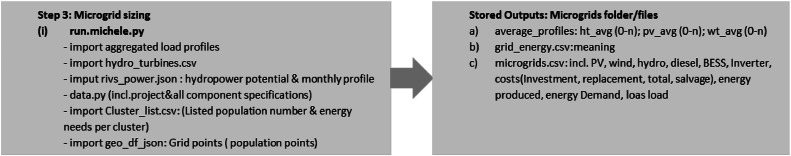
Summary of grid routing procedures and key outputs.

The computational efforts may increase when the technology sets are expanded and therefore risks the non-convergence of the model. The applied and detailed description of the mathematical algorithms (working procedures, dependencies’ constraint factors) can be found in [45,46]. The detailed list of relevant input files (configuration, load profile, land cover, imported_.csv, imported_subs, tilt angles and hydro turbines) is shown in Fig. 15, along with the key outputs delivered in step 3.

### NPC analysis and integrated optimisation (step 4)

The final step (step 4) targets the optimal electrification strategy for each cluster of communities, choosing between connections to an existing grid/substation or the off-grid hybrid microgrid designs (step 3). GISEle makes this choice based on comparing the computed LCOEs as expressed in Eq. (9) to finally selecting the least-cost electrification solutions. In this procedure, GISEle can calculate the two-electrification scenarios for each demand cluster. It does so as it considers the distance and costs for connecting the communities within the case area through medium voltage power lines to the nearest grid substation point. Hence, the procedure may advise all community clusters either to become off-grid hybrid microgrids or interconnected to the nearest national grid /substation or advice for some installed off-grid hybrid microgrids to be interconnected among them.

(9) $$LCOE=\frac{\sum_{t=1}^{T}\frac{C_{t}+O\&M_{i}+F_{t}}{{\left(1+r\right)}^{t}}\;}{\sum_{t=1}^{T}\frac{E_{t}}{{\left(1+r\right)}^{t}}}$$

**Fig. 16:**
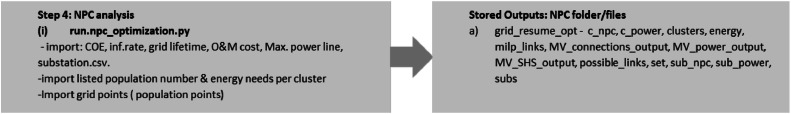
Summary of NPC analyses procedures and key outputs.

Being$\;C_{t}$ the capital expenditure, O&M the operation and maintenance cost of each technology, $F_{t}$ the fuel expenditure, E is the electrical energy generated, t is the year and T is the expected project lifetime. For isolated micro-grid systems, the LCOE is given by Eq. (10).

(10) $$LCOE_{mg}=\frac{C_{grid}}{\sum_{t=1}^{T}\frac{E}{{\left(1+r\right)}^{t}}}+LCOE_{gen}$$

where: mg and gen mean microgrid and generator respectively. C_grid_ is the capital cost of the internal grid as a function of $C_{u,v}$ weights (Eq. 5);

For considering the connection of a cluster's internal grid to the national grid (NG), additional electric line (MV/HV) costs with the closest substations are included as expressed by Eq. (11).

(11) $$LCOE_{HV}=\frac{C_{grid}+C_{con}}{\sum_{t=1}^{T}\frac{E_{max}}{{\left(1+r\right)}^{t}}}+LCOE_{NG}$$

where: C_con_ is the capital cost for the electric line connection between the cluster and the existing NG; E_max_ is the foreseen energy consumption from new connections at the maximum available capacity of the installed/connected infrastructure (Fig. 16).

### Computational time

The computation of the integrated methods (GISEle and RAMP) is conducted within a python environment, version 3.7 or higher. The models have been successfully tested in a computer with the following specifications: Intel(R) Core (TM) i7–8550 U CPU @ 1.80 GHz 1.99 GHz; RAM 16.0 GB; 64-bit operating system, x64-based processor. The grid routing procedure and NPC analysis are the most time-consuming step procedures, mainly due to the amount of input data required to be processed in the final MILP optimisation, where the Dijkstra algorithm has to run several times. For example, when more than one substation is loaded and long connection distances between nodes are detected. A time ranging from half to 1 hour or even days (in low-performance computers) may be required to run all the modeling procedures. Additional time may be required for other procedures such as the input data preparation and processing of the result output for reporting the maps. However, another time-consuming step concerns the process of generating load profiles and river flow rate estimations. This depends on the input details included (size of user classes, quantity of owned appliances, etc., in RAMP and the extension area of the watershed and other geospatial details in SWAT model, the generated outputs can be obtained after several hours of model running.

## Method validation

Several data can be used to run the different modules of the framework (see Appendix A, Table A1). The used data categories are explained breathily in the 2nd and 3rd columns, in Table A1. Previous and initial validation efforts can be found in [8] and specific data on case studies reported in [7] and [47](forthcoming) will be provided upon request*.*

## Limitations

The use of biomass resources as potential for power generation is not yet included in the microgrid sizing module/procedure.

## Ethics statements

The authors confirm that they have read and followed the ethical requirements for publication in MethodsX and that this work does not involve human subjects, animal experiments or any data collected from social media platforms.

## CRediT authorship contribution statement

**Berino Francisco Silinto:** Conceptualization, Methodology, Data curation, Visualization, Writing – original draft, Writing – review & editing. **Darlain Edeme:** Software, Methodology. **Silvia Corigliano:** Software, Methodology. **Aleksandar Dimovski:** Software, Methodology, Writing – original draft. **Marco Merlo:** Writing – review & editing. **Christian Zuidema:** Supervision, Writing – review & editing, Resources. **André Faaij:** Supervision, Methodology, Writing – review & editing.

## Declaration of competing interest

The authors declare that they have no known competing financial interests or personal relationships that could have appeared to influence the work reported in this paper.

## Figures and Tables

**Table:** **Table A.1**Input parameters and description including their gathering sources, are required to be processed or directly input in the framework-integrated procedures and tools.

Parameters	Data type	Description/resolution	Source data/reference	GISELE	RAMP	SWAT
Administrative boundaries	Vector layer (polygon)	Used to define the boundaries of the study area	[15,16]	x		x*
Population and density distribution	Raster layer [people/m^2^]	Amount of people living in a grid cell (100 m) – used in the clustering procedure	High-resolution settlement layer database [[17], [18]] https://www.worldpop.org/data/www.ine.gov.mz	x	x	
Buildings (households, health, school, water facilities, etc.,)	Vector layer	Detailed information on existing infrastructure/facilities. This is a country-specific provided information (100 m)	[16] Google satellite imageries	x	x	
Wind speed	Raster layer [m/s]	Meteorological parameter to assess wind power potential (1000 m)	https://globalwindatlas.info/ https://www.worldclim.org	x		x
Global Horizontal Irradiation (GHI)	Raster layer [kWh/m^2^/year]	Meteorological parameter to assess solar irradiation potential (2–6km/ 90(TMY); 250 m Global atlas)	https://globalsolaratlas.info/	x		x
Temperature (monthly mean; mean; min/max)	Raster layer [ °C]	Meteorological parameter to assess temperature potential (1000 m)	https://www.worldclim.org	x		x
Rainfall/Precipitation (monthly)	Raster layer [mm/month]	Meteorological parameter to assess rainfall potential (1000 m)	https://www.worldclim.org/	x		x
Relative Humidity	Raster [fraction]	Meteorological parameter to assess relative humidity potential (1000 m)	https://www.worldclim.org/			x
Land use/cover/ GLC 2000	Raster layer [m]	Raster layer providing information on land cover, with 10 different land cover types provided at high resolutions (10/30 m)	https://www.diva-gis.org/ [19]	x		x
Digital Elevation Model (DEM-SRTM)	Raster layer [m]	A mix of raster layers provides information on the elevation from which the slope layer is computed. (30m/90 m)	[20,19],	x		x
Wind and solar		Meteorological parameters to assess wind and solar radiation energy potential	Internal routine: [33,35]	x		
River flow rates	[m^3^/s]	This can be derived from the SWAT model. Used to estimate the hydropower potential	https://swat.tamu.edu/ and [50,51]	X		
Global River Network (HydroSHEDS)	Vector layer (Polyline)	Provide information on the average river flow rate, penalised crossing of large rivers	https://www.hydrosheds.org.v [21]			x
Global Streamflow Characteristics Dataset (GSCD)	Raster		[52,53]			x
Energy demand and load profiles	(.csv)	Based on socio-economic and demographics, appliances and usage patterns collected from surveys. Define elements for definitions of demand patterns Derived from the RAMP model:	https://github.com/SESAM-Polimi/RAMP_multiyear [29,10]	x		
Existing/planned transmission& distribution lines	Vector line/point/shapefile	Used to access whether is possible to extend the grid or not	https://energydata.info/n.a. www.edm.co.mz	x		
Road network	Vector layer/polylines	A layer with information on existing rod networks. Used in the creation of Pfs and cost surface areas and grid routing	https://www.openstreetmap.org/	x		
Water bodies(lakes)	Vector layer	Polygon	https://www.openstreetmap.org	x		x
Protected areas	Vector layer/polylines	A layer with information on restriction zones: Used in the creation of Pfs and cost surface area	[23,24]	x		
Hydrologic soil type	Raster layer with 8 soil groups	Used for evaluating soil types in the process of defining HRUS	https://daac.ornl.gov/			x
National diesel prices	l/h(l/day)	To update (2016)	Pump price for diesel fuel (US$ per litre) - Mozambique | Data (worldbank.org)	x		
Component costs: PV panels. Wind turbines, hydro turbines, batteries, converters, Generators etc	RE Technology /component costs and performance	Gathered from surveys from National Energy supporting & Literature	Surveys (FUNAE) [54,55]	x		

**Table:** 

#%% Definition of the inputs ''' Public Services load demands ''' from core import User, np User_list = [] ''' This example input file represents a single community baseload, providing most of the possibilities ensured by RAMP for input definition, including specific modular duty cycle. ''' #Create new user classes # Public User Class Type
#1: Load demand for Health Center HCenter = User("HCenter_HC",2) User_list.append(HCenter) #Creation of new appliances HC_outdoor_bulb = HCenter.Appliance(HCenter,8,25,2780,0,780, 'yes', flat = 'yes') HC_outdoor_bulb.windows([0,360],[1050,1440],0) HC_indoor_bulb = HCenter.Appliance(HCenter, 18,20,2690,0.2,480) HC_indoor_bulb.windows([360,1320],[0,0],0.35) HC_indoor_tubes = HCenter.Appliance(HCenter, 7,30,1540,0.2,480) HC_indoor_tubes.windows([360,1320],[0,0],0.35) HC_phone_charger = HCenter.Appliance(HCenter,6,10,2360,0.2,60, occasional_use = 0.75) HC_phone_charger.windows( [0,1440], [0,0],0.35) HC_sterilizer = HCenter.Appliance(HCenter,2120,1120,0.2,60, occasional_use = 0.75) HC_sterilizer.windows([480,1020],[0,0],0.35) HC_incubator = HCenter.Appliance(HCenter,2360,1,1440,0.3,720, occasional_use = 0.75) HC_incubator.windows([0,1440],[0,0],0) HC_PCcomp = HCenter.Appliance(HCenter,1100,3540,0.1,60) HC_PCcomp.windows( [8 × 60+30,12×60+30], [15×60,18×60],0.35, [20×60,24×60]) HC_Fridge = HCenter.Appliance(HCenter,2300,1,1440,0,30, 'yes',3) HC_Fridge.windows( [0,1440], [0,0]) HC_Fridge.specific_cycle_1(250,20,5,10) HC_Fridge.specific_cycle_2(250,15,5,15) HC_Fridge.specific_cycle_3(250,10,5,20) HC_Fridge.cycle_behaviour([580,1200],[0,0],[420,579],[0,0],[0,419],[1201,1440]) HC_CeilingFan = HCenter.Appliance(HCenter,4,80,2420,0.1,15) HC_CeilingFan.windows([420,540],[1020,1260],0.35)

## Data Availability

Data will be made available on request.

## Appendix A – Input datasets required for the different tools and modules

Starting from the administrative boundaries vector layer of the study area, most of the required input data sets can be extracted from the literature and officially published reports from the governmental, non-governmental, and international agencies dealing with or supporting the rural electrification sectors [48]. Conversely, additional datasets can be collected through specific field surveys and used to derive estimates on demand profiles within the RAMP tool. Furthermore, lacking geospatial data (often in most developing regions) can be retrieved from openly and publicly available online data sources in GIS-based and remote sensing data formats derived from satellite imageries. These include the population [18,49], the digital elevation maps (DEM), slope, land use (LU)/land cover (LC), and gridded weather (wind and solar) datasets [35], etc., which are key inputs required to initialise GISELE analysis and to derive terrain characteristics through GISELE's internal automated procedure database. Moreover, some of the listed datasets are also inputs to the proposed SWAT model [40] for computing the river flow rate estimates required in GISEle's new upgraded code that sizes hydropower generation capacities.

## Appendix B

The example below illustrates a Python coding for estimating the load demand profile of a single rural user: Health services. The “#Creation of new appliances” line is where we add each type and number of adopted appliances and other details in terms of nominal power absorbed by the appliance, usage patterns: time windows associated to specific duty cycles, etc.

## Footnotes

1 A free and open-source interactive tool for field data collection. Available in a web application which allows design questionaires/survey forms and deploy and download submited data (https://www.kobotoolbox.org/).

2 QGIS-Quantum GIS (https://www.qgis.org/en/site/).

3 Vectors are mostly useful for precise identification and representation of geographic boundaries and features with a high degree of accuracy and for conducting network analyses and topological operations. In this study, they mainly apply to roads, grids and waterways. Rasters are mostly suitable for representing continuous and detailed data variables where each geographic location has a specific data value. In this study, they mainly apply topopulation, elevation, land use and soil. Appendix Table A of the manuscript presents the datasets used.

4 Longitude, latitude geographical coordinates.

5 Cost surface - represents a realistic least-cost per pixel for building an electric line. The final line cost/kilometre is a location-dependent “penalty factor-Pf” to be multiplied with. Cost-surface raster files can also be used as constraint indicators for sitting energy plants.

6 Global land cover database for the year 2000.

7 Clustering is an iterative method for processing a set of similar data points/objects and grouping them together on the basis of their linkage in relation to the other similar or dissimilar points/objects [56].

8 Maximum distance between two sample clusters for one to be considered as its neighborhood where the Euclidean distance is used as the distance metric between points [27].

9 Based on an hierarchic structure, this procedure can model two different types of cable: (i) Main branch/feeder - constitution the backbone of the grid and (ii) Collaterals - originating from the main feeder and connecting all the other users.

10 From standard wind turbines and PV modules.

11 Heads along the river are computed using DEM layer based on a linear regression model along the river stream sampled at each 100/200m.
